# Protective efficacy of 2-PAMCl, atropine and curcumin against dichlorvos induced toxicity in rats

**DOI:** 10.2478/v10102-012-0001-x

**Published:** 2012-03

**Authors:** Preeti Yadav, Sunil E. Jadhav, Vinesh Kumar, Kirtee K. Kaul, Satish C. Pant, Swaran J.S. Flora

**Affiliations:** 1 Division of Pharmacology & Toxicology, Defence Research and Development Establishment, Jhansi Road, Gwalior, India; 2 School of Studies in Botany, Jiwaji University, Gwalior, India

**Keywords:** Dichlorvos toxicity, 2-PAM.Cl, curcumin, atropine, oxidative stress

## Abstract

The effect of 2- pyridine aldoxime methyl chloride (2-PAMCl) and atropine with or without curcumin was investigated in dichlorvos (2,2-dichlorovinyl dimethyl phosphate; DDVP) induced toxicity in rats. Rats were exposed to DDVP (2 mg/kg sub-cutaneously) once daily for the period of 21 days. Post DDVP exposure, rats were further treated with 2-PAMCl (50 mg/kg intramuscular, once daily) + atropine (10 mg/kg, i.m. once daily) with or without curcumin (200 mg/kg; oral; once daily) for further 21 days. We observed a significant increase in lipid peroxidation (LPO), reactive oxygen species (ROS), oxidized glutathione (GSSG), while there was a significant decrease in antioxidant enzymes, brain acetylcholinesterase (AChE) and 5-hydroxy tryptamine (5-HT) activity on DDVP exposure of rats. These alterations were restored significantly by co-administration of 2-PAMCl + atropine in DDVP exposed rats. Curcumin when co-supplemented with 2-PAMCl + atropine also significantly protected serum aspartate aminotransferase (AST) and restored brain AChE activity and 5-HT level in animals sub-chronically exposed to DDVP. Histopathological observations along with biochemical changes in rat blood and tissues revealed significant protection offered by 2-PAMCl + atropine against DDVP. The results indicate that DDVP-induced toxicity can be significantly protected by co-administration of 2-PAMCl + atropine individually, however, curcumin co-supplementation with 2-PAMCl + atropine provides more pronounced protection, concerning particularly neurological disorders.

## Introduction

Organophosphorous compounds (OP) have been used as pesticides (chlorpyrifos, paraoxon, DDVP, *etc.*) and as chemical warfare nerve agents (sarin, tabun, agent VX, *etc.*). The irreversible binding to and subsequent inactivation of acetylcholinesterase, an enzyme that normally catalyzes hydrolysis of acetylcholine (ACh) at neuromuscular junctions and other cholinergic synapses, is generally believed to be the major mechanism of their toxicity (Shenouda *et al.*, 2009).The subsequent accumulation of ACh in the cholinergic clefts causes overstimulation of the peripheral as well as the central cholinergic nervous system resulting in clinical manifestations in the form of acute cholinergic crisis (Taylor, 2001; Savolainen, 2001). Dichlorvos has been widely and effectively used throughout the world in agriculture for controlling insects that damage crops. For the treatment of nerve agent/OP poisoning several antidotes have been evaluated and the currently recommended drugs are atropine and pralidoxime chloride. Pralidoxime chloride is an oxime, which reactivates the inhibited AChE and restore function at the neuromuscular junction (Taylor, 2001). Pralidoxime has therapeutic efficacy against acute toxicity of dichlorvos (DDVP) (Zhu *et al.*, 2005). The clinical experience with the use of PAM-2 iodide given with atropine and diazepam was extremely favorable in the treatment of the victims of the Tokyo Sarin attack in 1995 (Stojiljkovic and Jokanovic, 2005). However, PAM-2 should not be recommended as the drug of choice in poisoning with warfare nerve agents due to its lack of efficacy against Tabun and Soman (Kassa, 2005). Certain oximes when used in combination with atropine were found to be more effective than atropine alone in organophosphate toxicity (Weissman and Raveh, 2008). Atropine is usually prepared following extraction from the plants *Atropa belladonna* (deadly nightshade), *Datura stramonium* (Jimson weed) or *Duboisia myoporoides* (McEvoy 2002). Atropine blocks the effect of excess acetylcholine at muscarinic receptors in the brain and peripheral tissues due to inhibition of the enzyme acetylcholinesterase (AChE).

Besides the aforesaid manifestations of its toxicity, OP induced oxidative stress further contributes to tissue damage. However, reports on anti-oxidative effects of 2-PAMCl and atropine in OP induced toxicity are scanty. In recent years, accumulating evidence has supported the protective effects of phenolic antioxidants from medicinal plants against oxidative stress mediated disorders (Soobratee *et al.* 2005). Several studies have indicated a beneficial role of curcumin in terms of its antioxidant, anti-tumorigenic, and anti-inflammatory action (Bengmark, 2006). It is also known to scavenge free radicals. The beneficial effects of *Curcuma longa* have been postulated to be due to the phenolic yellow pigments of turmeric, curcuminoids, along with the major compound curcumin (Miquel *et al.* 2002).

Despite the above-mentioned beneficial effects of curcumin, its efficacy in OP toxicity particularly in dichlorvos induced toxicity, has not yet been studied. The present study was designed to evaluate (i) DDVP-induced oxidative stress in blood and soft tissues in rat, (ii) beneficial effects of curcumin in combination with 2-PAMCl and atropine in preventing DDVP-induced oxidative stress, and (iii) histopathological alterations in liver and brain of rats.

## Materials and methods

Experiments were performed on male Wistar rats weighing 120±10 g. The animals were obtained from the animal facility of Defence Research and Development Establishment. All animals received humane care in compliance with the guidelines of the “Institutional Animal Ethics Committee (IAEC)”. The Animal Ethics Committee of DRDE, Gwalior, India, approved the protocols for the experiments. The animals were housed in polypropylene cages on dust-free and autoclaved paddy husk and provided with standard pellet diet (Ashirwad Feeds, India) and water *ad libitum.* The animals were maintained under standard conditions of temperature and humidity with alternating 12 h light/dark cycles.

Forty male Wistar rats were equally divided into four groups of 10 animals each. For the first 3 weeks, DDVP was administered subcutaneously once daily and post-treatment with 2-PAMCl and atropine alone and in combination with curcumin was carried out for further 3 weeks. Details of groups and different treatments are given below.

Group I: Normal Control (Drinking water)

Group II: DDVP (2 mg/kg body weight subcutaneously) once daily for three weeks

Group III: DDVP (2 mg/kg body weight subcutaneously) once daily for three weeks and intramuscular administration of 2-PAMCl and atropine (50 and 10 mg/kg body weight, respectively) once daily for further three weeks (4 to 6 weeks)

Group IV: DDVP (2 mg/kg body weight subcutaneously) once daily for three weeks and 2-PAMCl + atropine (as in group III) and oral administration of Curcumin (200 mg/kg body weight dissolved in groundnut oil) once daily for three weeks (4 to 6 weeks).

Rats were weighed weekly throughout the experimental period. After administration of the last dose, the animals were given 24 hours of rest and were then sacrificed under light ether anesthesia. Blood was collected in heparinized tubes. For the collection of serum, blood was collected in non-heparinized tubes. Liver and brain were dissected out, rinsed in cold saline, blotted, weighed and used for various biochemical variables. Liver and brain from each rat were processed immediately for biochemical estimation as well as for histopathology. Five animals from each group were used for biochemical and histopathological observations.

Analysis of blood GSH concentration was performed with the method described by Ellman *et al.* (1959) and modified by Jollow *et al.* (1974). The generation of ROS was performed as described by Socci *et al.* (1999). TBARS was measured by the method of Ohkawa *et al.* (1979). Alanine amino transferase (ALT) and aspartate amino transferase (AST) activities in serum were assayed according to the method of Reitman and Frankel. (1957). Liver GSH and GSSG levels were measured as described by Hissin and Hilf. (1976). Liver SOD activity was assayed by the method of Kakkar *et al.* (1984). Catalase activity was assayed following the procedure of Aebi (1984). The frozen brain tissue samples were weighed and homogenized in acidified butanol. 5-hydroxytryptamine (5-HT) was estimated according to the procedure of Jacobwitz and Richardson (1978). A 10% brain homogenate (w/v) was prepared in 0.25 M sucrose. Activity of acetylcholinesterase (AChE) in brain was determined according to the method of Ellman *et al.* (1961) using acetylthiocholine as substrate. The activity of AChE was measured at 412 nm and its unit is expressed as nmol/min/mg protein.

Liver and brain tissues fixed in Bouin's fluid were dehydrated through a graded series of alcohol clearing in toluene using automatic tissue processor (Leica TP 1020) and embedded in paraffin. Liver tissues were sectioned at 5 μm thickness and brain sections were taken at 12 μm, which was followed by hematoxylin and eosin staining (H&E stain), and observed under light microscope (Leica, DMLB).

All data were expressed as means±SEM. Data were analyzed statistically using analysis of variance followed by Bonferroni test. Statistical significance was set at *p<*0.05. Data analysis was performed with GraphPad InStat (GraphPad Software Inc., 5755 Oberlin Drive, #110, San Diego, CA 92121, USA).

## Results

### Effect of 2-PAMCl and atropine with or without curcumin on hematological variables in DDVP exposed rats

Table 1 shows the effect of co-administration of curcumin, 2-PAMCl and atropine after DDVP exposure of rats. There were no significant changes in any of the hematological variables in rats from the treated groups.

**Table 1:** Effect of co-administration of 2-PAMCl, atropine and curcumin on hematological variables in DDVP intoxicated rats.

Parameters	Control	DDVP	DDVP + 2-PAMCl + Atropine	DDVP + 2-PAMCl Atropine + Curcumin +
WBC	22.7±1.28	27.3±2.52	26.6±3.23	21.9±3.27
RBC	8.3±0.10	8.4±0.10	8.3±0.10	16.5±1.05
HGB	15.2±0.13	14.8±0.33	14.7±0.21	14.1±0.21
HCT	45.6±0.26	45.4±0.57	45.3±1.11	44.1±0.64
MCV	54.6±0.85	54.4±1.23	55.0±0.97	53.8±0.49
MCH	18.2±0.27	17.6±0.59	17.6±0.31	17.2±0.18
MCHC	33.3±0.25	32.5±0.68	32.1±0.37	32.0±0.20
PLT	736±78.2	703±39.78	784±53.49	710±24.49

Abbreviation used and units: WBC-white blood cells (×10^3^ μl); RBC-red blood cells (×10^6^ μl); Hemoglobin (g/dl); hematocrit (%); MCV-mean cell volume (fl); MCH-mean cell hemoglobin (pg); MCHC-mean cell hemoglobin concentration (g/dl); PLT – platelets (×10^3^μl). Values are mean ±S.E; n=5.

### Effect of 2-PAMCl and atropine with or without curcumin on serum biochemical variables in DDVP exposed rats

**Figure 1:**
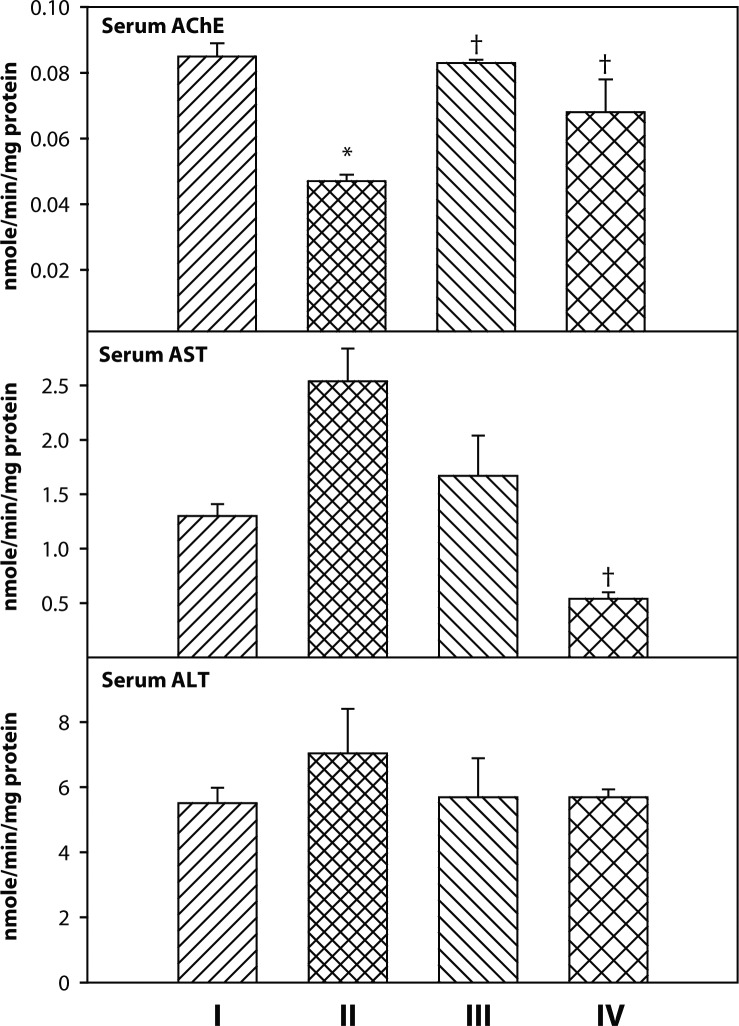
Effect of co-administration of 2-PAMCl, atropine and curcumin on serum biochemical variables in DDVP intoxicated rats. **I**: Control, **II**: DDVP, **III**: DDVP + 2-PAMCl and atropine, **IV**: DDVP + 2-PAMCl and atropine + curcumin. Abbreviations used: AChE-Acetylcholinesterase, AST-Aspartate transaminase; ALT-Alanine transaminase. Values are mean±S.E.; n=5. ^*^Significantly (*p<*0.05) different as compared to control rats. ^†^Significantly (*p*<0.05) different as compared to DDVP exposed rats.

Exposure to dichlorvos alone significantly reduced serum AChE level compared to control rats (Figure 1). There was a significant increase in serum AChE activity following co-administration of 2-PAMCl + atropine or 2-PAMCl + atropine + curcumin with DDVP compared to the DDVP alone group. Serum AST activity recovered significantly in rats treated with 2-PAMCl + atropine + curcumin post DDVP exposure. There was no change in serum ALT level in any of the experimental groups.

### Effect of 2-PAMCl and atropine with or without curcumin on blood ROS and GSH level in DDVP exposed rats

**Figure 2:**
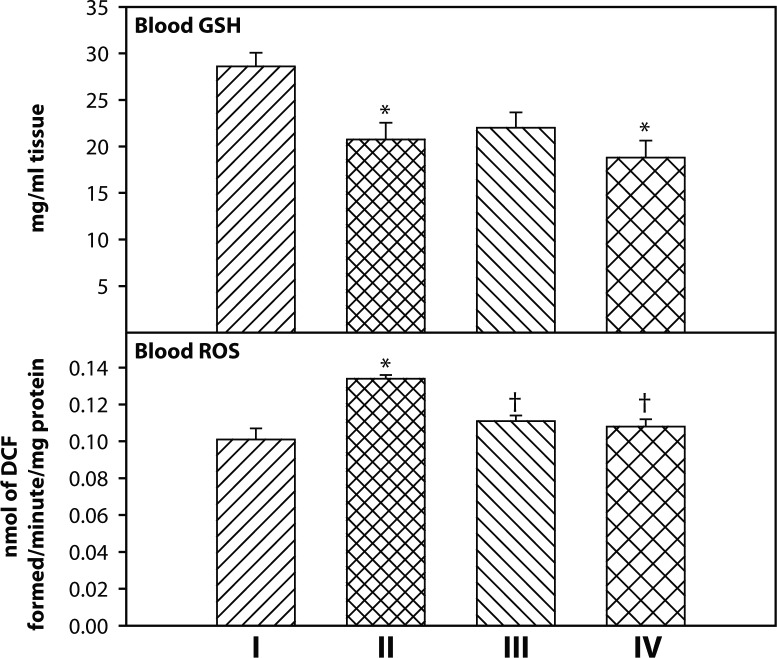
Effect of co-administration of 2-PAMCl, atropine and curcumin on blood ROS and GSH level in DDVP exposed rats. **I**: Control, **II**: DDVP, **III**: DDVP + 2-PAMCl and atropine, **IV**: DDVP + 2-PAMCl and atropine + curcumin. Abbreviations used: ROS-reactive oxygen species; GSH-reduced glutathione. Values are mean±SE. n=5. ^*^Significantly (*p<*0.05) different as compared to control rats. ^†^Significantly (*p<*0.05) different as compared to DDVP exposed rats.

Blood ROS and GSH levels in different groups are presented in Figure 2. Blood ROS level was significantly (*p<*0.05) higher in the DDVP group compared to the control group. However, blood ROS level decreased significantly (*p<*0.05) after treatment with 2-PAMCl + atropine and 2-PAMCl + atropine + curcumin. There was a significant (*p<*0.05) decrease in blood GSH level in DDVP alone and DDVP along with 2-PAMCl + atropine + curcumin treatment groups compared to the control group.

### Effect of 2-PAMCl and atropine with or without curcumin on liver biochemical variables in DDVP exposed rats

**Figure 3:**
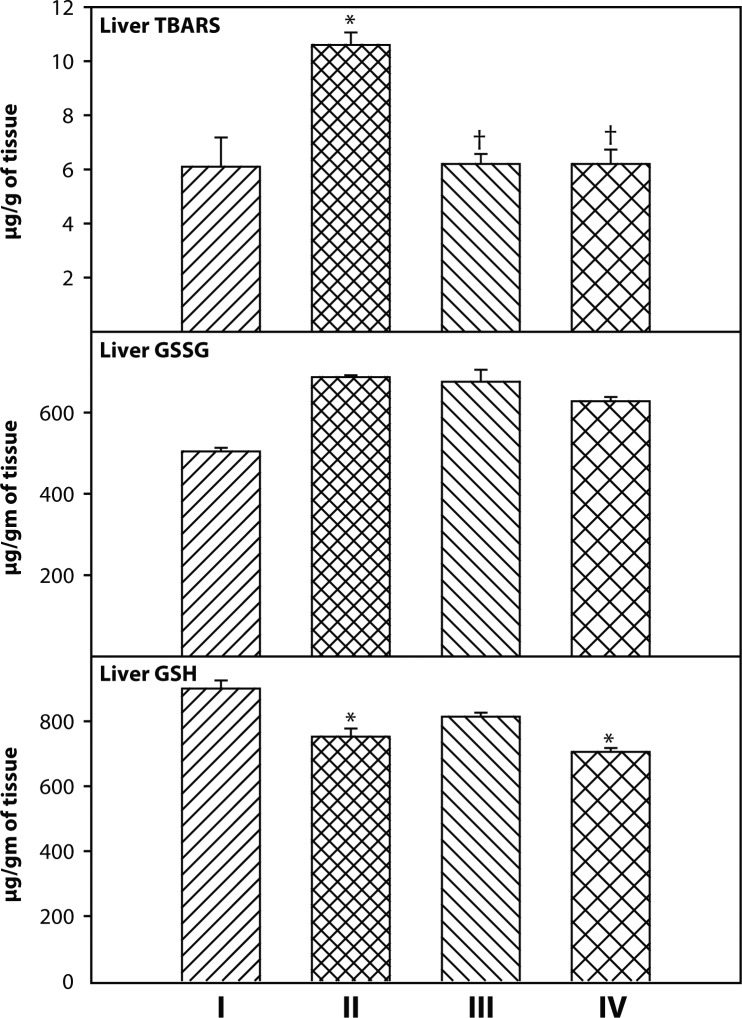
Effect of co-administration of 2-PAMCl, atropine and curcumin on liver GSH, GSSG and TBARS levels in DDVP intoxicated rats. **I**: Control, **II**: DDVP, **III**: DDVP + 2-PAMCl and atropine, **IV**: DDVP + 2-PAMCl and atropine + curcumin. Abbreviations used: GSH-reduced glutathione; GSSG-oxidized glutathione; TBARS-thiobarbituric acid reactive substances. Values are mean±SE; n=5. ^*^Significantly (*p<*0.05) different as compared to control rats. ^†^Significantly (*p<*0.05) different as compared to DDVP exposed rats.

The level of TBARS was significantly elevated in DDVP exposed groups as compared to the control group. However, treatment with 2-PAMCl + atropine alone and in combination with curcumin restored TBARS level towards normal as shown in Figure 3. Liver GSH level was significantly depleted in groups exposed to DDVP and supplementation of curcumin with 2-PAMCl + atropine recovered the GSH level. There were no changes in liver GSSG levels in any of the groups studied (Figure 3).

### Effect of 2-PAMCl and atropine with or without curcumin on liver SOD and catalase levels in DDVP exposed rats

**Figure 4:**
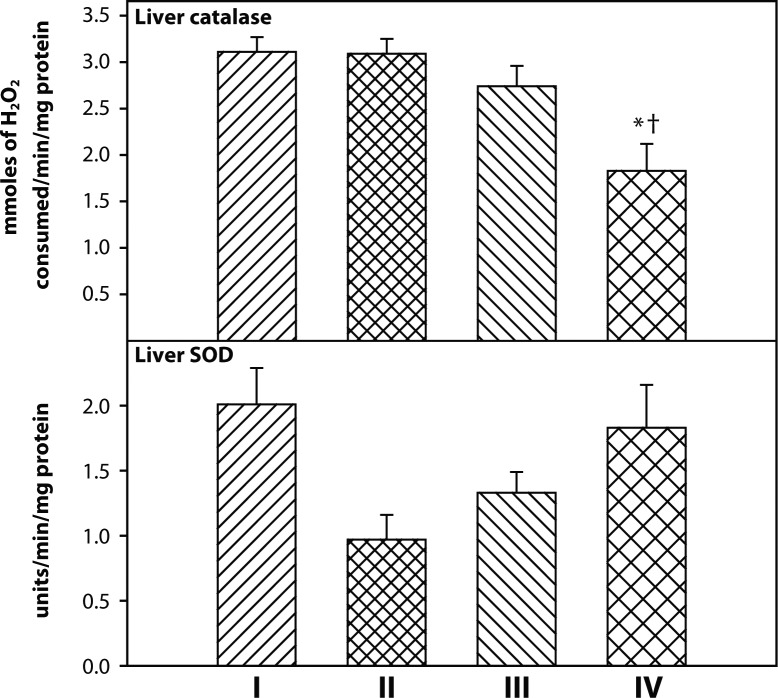
Effect of co-administration of 2-PAMCl, atropine and curcumin on liver SOD and catalase levels in DDVP intoxicated rats. **I**: Control, **II**: DDVP, **III**: DDVP + 2-PAMCl and atropine, **IV**: DDVP + 2-PAMCl and atropine + Curcumin. Abbreviation used: SOD-superoxide dismutase. Values are mean±SE; n=5. ^*^Significantly (*p<*0.05) different as compared to control rats. ^†^Significantly (*p<*0.05) different as compared to DDVP exposed rats.

Liver catalase activity in rats exposed to DDVP and treated with 2-PAMCl + atropine + curcumin was significantly lower as compared to control and DDVP alone exposed groups (Figure 4). There were no significant changes in liver SOD activity.

### Effect of 2-PAMCl and atropine with or without curcumin on brain AChE activity and 5-HT level in DDVP exposed rats

**Figure 5:**
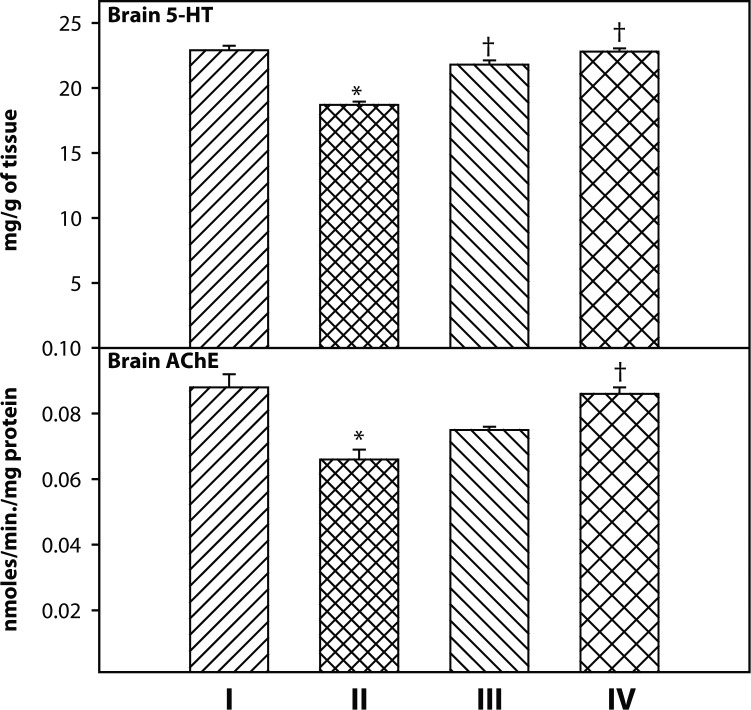
Effect of co-administration of 2-PAMCl, atropine and curcumin on brain biochemical variables in DDVP intoxicated rats. **I**: Control, **II**: DDVP, **III**: DDVP + 2-PAMCl and atropine, **IV**: DDVP + 2-PAMCl and atropine + curcumin. Abbreviations used: AChE-acetylcholinesterase; 5-HT-5-hydroxytryptamine. Values are mean±SE; n=5. ^*^Significantly (*p<*0.05) different as compared to control rats. ^†^ Significantly (*p<*0.05) different as compared to DDVP exposed rats.

A significant decrease in brain AChE and 5-HT level was observed in DDVP exposed rats compared to the control group (Figure 5). However when compared with DDVP exposed rats, there was a significant recovery in brain AChE activity in the group treated with 2-PAMCl + atropine + curcumin. 5-HT levels decreased marginally in the DDVP alone group and recovered significantly when treated with 2-PAMCl + atropine alone and in combination with curcumin after DDVP exposure.

### Effect of 2-PAMCl and atropine with or without curcumin on histopatholocurcumin on histopathological changes in liver and brain of rats intoxicated with DDVP

**Figure 6:**
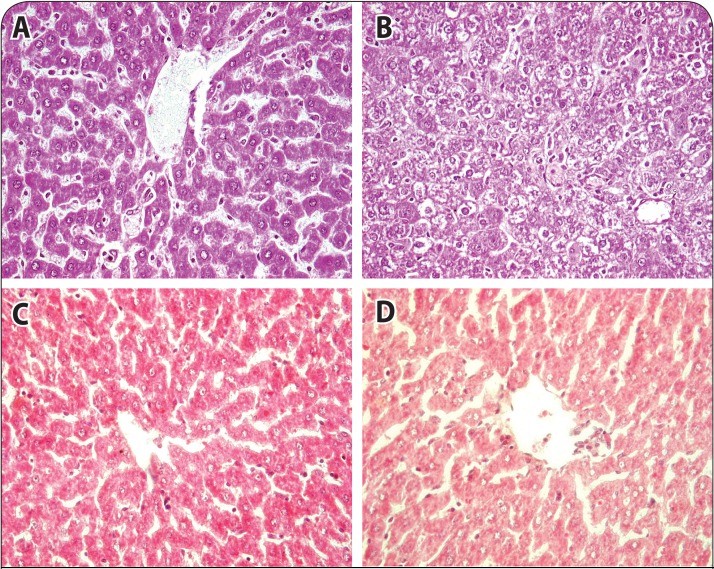
Photomicrographs of liver sections of control, DDVP exposed and treated rats, H&E 40×. **A:** Control liver showing normal lobular architecture with hepatocytes arranged in cords encircling the central canal. **B:** DDVP exposed rat liver showing cytoplasmic clumping, hyperactivation of Kupffer cells, vacuolation, necrosis and ballooning of hepatocytes, **C** and **D:** 2-PAMCl and atropine and 2-PAMCl and atropine + curcumin (200 mg/kg) treatment after DDVP exposure showing few mild hyperactive Kupffer cells and necrotic cells in hepatic parenchyma.

**Figure 7:**
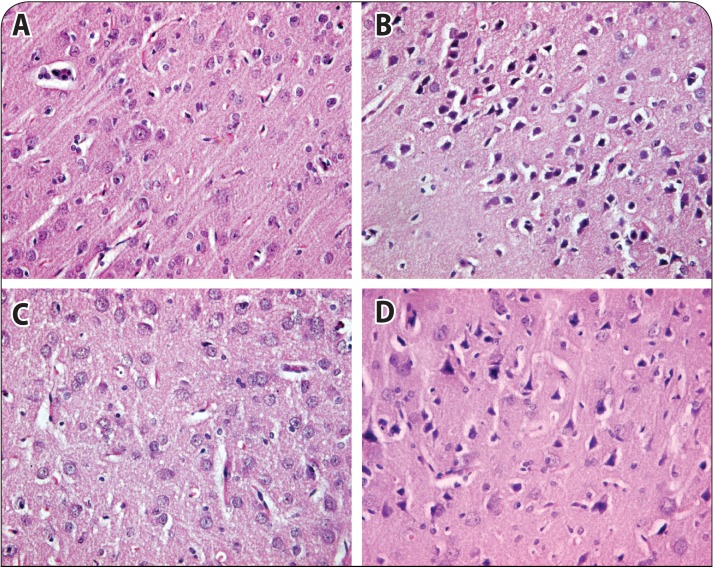
Photomicrographs of brain histopathology of control, DDVP exposed and treated rats, H&E 40×. **A:** Control rat brain showing normal neuroglial cells arranged in several layers. **B:** DDVP exposed rat brain showing chromatolysis of nuclear material, gliosis and pyknotic neurons in cortex. **C:** Rat brain with DDVP + 2-PAMCl and atropine showing mild lesions of neuronal damage with moderate gliosis. **D:** 2-PAMCl + atropine + curcumin (200 mg/kg) treatment after DDVP exposure showing mild gliosis and chromatolysis along with minimal lesions of neuronal degeneration.

The histopathological findings in rat liver following DDVP exposure are given in Table 2 and shown in Figure 6. The liver of control rats showed normal lobular architecture with hepatocytes arranged in cords encircling the central canal. Liver sections of DDVP exposed rats showed cytoplasmic clumping, hyperactivation of Kupffer cells with foamy cytoplasm, hepatocellular vacuolation, necrosis of hepatocytes, basophilia in nuclei representing karyorrhexis and hepatocytes showing karyolysis along with ballooning. Rats treated with 2-PAMCl + atropine post DDVP exposure showed a few hyperactive Kupffer cells and necrotic cells in the hepatic parenchyma. However, mild cytoplasmic clumping, hyperactive Kupffer cells, hepatocyte vacuolation, necrosis, and inflammatory foci were observed in sections of the liver of rats treated with 2-PAMCl + atropine + curcumin after DDVP exposure. Neuronal integrity was assessed in the cortex (Table 3; Figure 7) and hippocampus of the brain in control and treated rats. The cortex region of control rat showed various types of normal neuroglial cells arranged in several layers. The brain of rats treated with DDVP showed moderate to severe neuronal degeneration and proliferation of glial cells along with moderate chromatolysis and inflammation of glial cells. Rats exposed to DDVP and treated with 2-PAMCl + atropine showed minimal to mild lesions of neuronal tissue. However mild gliosis and chromatolysis along with minimal lesions of neuronal degeneration were observed in brain section of rats treated with 2-PAMCl + atropine + curcumin after DDVP exposure. The hippocampal region of control rats showed normal cellular composition in all three layers (Figure 2). However subcutaneous DDVP exposure of rats resulted in severe chromatolysis and necrosis of Purkinje fibers of the hippocampal region. The severity of lesions in DDVP exposed rats treated with 2-PAMCl + atropine was minimized compared to DDVP exposed rats, whereas in 2-PAMCl + atropine + curcumin treated animals mild neuronal lesions were seen (Table 3).

**Table 2:** Effect of co-administration of 2-PAMCl, atropine and curcumin on liver histopathological changes in rats intoxicated with DDVP.

Lesions	Control	DDVP	DDVP + 2PAMCl + atropine	DDVP + 2PAMCl + atropine + curcumin
Clumping of cytoplasm	+	+ + + +	−	+ +
Hyperactivation of Kupffer cells	−	+ +	+ +	+ +
Hepatocyte vacuolation	−	+ + + +	−	+ +
Necrosis/Apoptosis	+ /−	+ + +	+	+ +
Infiltration of inflammatory cells	−	+ + + +	−	+ +
Karyorhexis/Karyolysis	−	+ + +	−	+
Ballooning of hepatocytes	+ /−	+ +	−	+ +

N=6. – Nil; + , Minimal (<12%); + + , Mild (<22%); + + + , Moderate (<45%) and + + + + , Severe (>45%).

## Discussion

The widespread use of pesticides produces a number of serious health effects affecting both humans and animals. The results of the present study suggests a beneficial role of curcumin with or without 2-PAMCl + atropine in DDVP exposed rats by providing effective protection to a number of biochemical variables and parameters indicative of oxidative stress, particularly in rat blood and tissues. Liver and brain are the major target organs of DDVP exposure as its metabolism occurs in the liver and the brain is particularly susceptible to oxidative damage due to the high utilization of inspired oxygen.

The liver plays an imperative role in the metabolism and biotransformation by which toxic compounds get converted into less harmful products (Hodgson, 2004).

Transaminases (ALT and AST) were used as important biomarkers for determining hepatotoxicity. ALT is a cytosolic enzyme, more specific for the liver. While AST is the mitochondrial enzyme, predominantly found in the liver, skeletal muscles and kidneys. The increase in their activity occurs mainly due to leakage of transaminases from liver cytosol into the blood stream, indicating liver damage or dysfunction. This increase may occur due to the formation of reactive oxygen species after dichlorvos exposure. Our results showed a pronounced increase in serum AST and ALT activity indicating hepatotoxicity. These results coincide with previous studies (Dwivedi & Flora, 2011; Dwivedi *et al.*, 2010; Manal *et al.*, 2008) that showed significant increases in the activity of transaminases in rats and humans exposed to organophosphate pesticides (OP). Treatment with 2-PAMCl and atropine had no effect on liver AST level, but co-administration of curcumin along with 2-PAMCl and atropine resulted in decreased liver AST activity, indicative of hepato-protecitve action of curcumin in DDVP toxicity (Ataman *et al.*, 2010). Fu *et al.* (2008) reported hepatoprotective action of curcumin in various animal models of liver injury.

Acetylcholinesterase (AChE) inhibition is a well known mechanism of organophosphate pesticide toxicity. Organophosphates inhibit AChE by phosphorylating serine residues at its active site resulting in accumulation of acetylcholine (ACh) in the synapse that produces cholinergic effects. In the present study, AChE activity was significantly inhibited by dichlorvos treatment, indicating OP poisoning (Ojha *et al.*, 2011). Celik and Isik (2009) also demonstrated that administration of sub-acute dichlorvos inhibited AChE and butylcholinesterase activities in various tissues of rats.

Biogenic amines are known to be highly targeted by organophosphates in the developing brain (Slotkin, 2004, 2005). The presented results revealed that 5-HT level decreased non-significantly in DDVP exposed rats. However, the level of brain 5-HT and AChE recovered marginally towards normal in both treated groups. The most pronounced beneficial effect was observed in rats treated with 2-PAMCl + atropine + curcumin suggesting their neuroprotective property.

Oxidative stress is reported to be one of the important mechanisms involved in OP toxicity (Llopis *et al.*, 2003). Being lipophilic in nature, OP interact with cells through biomembranes rich in polyunsaturated fatty acids, thus oxidatively damaging them, known as lipid peroxidation (Mittal and Flora, 2006). We observed increased TBARS level in the present study predominantly in animals given DDVP (Dwivedi *et al.*, 2010). The effect of curcumin on lipid peroxidation has been widely studied in various models. It was found to inhibit lipid peroxidation in rat liver microsomes, erythrocyte membrane and brain (Reddy and Lokesh, 1994).

Oxidative stress occurs usually with excessive generation of free radicals followed by a parallel depletion of antioxidant enzymes like GSH. Reduced GSH and its metabolizing enzymes provide the major defense against ROS induced cellular damage (Celik and Suzek, 2008).Organophosphate appears to disturb this key cellular pathway perhaps by disturbing mitochondrial metabolism, as suggested by Delgado *et al.* (2006). In the present study, we observed depleted levels of antioxidant enzymes and elevated levels of ROS and TBARS following DDVP exposure in rats. Curcumin is known to exhibit a strong antioxidant activity and is a potent scavenger of free radicals such as superoxide anion radicals, hydroxyl radicals and nitrogen dioxide radicals (Maheshwari *et al.*, 2006; Anand *et al.*, 2008). Curcumin treatment improved the levels of antioxidant enzymes, mainly GSH, SOD and catalase (Tirkey *et al.*, 2005).

In the present study, histopathological findings supported the results of biochemical variables. Histology of the liver showed signs of hepatocellular degeneration, vacuolation, inflammation and pyknosis. Histology of the brain showed severe degenerative and necrotic changes, similarly as reported by Abdollahi *et al.* (2004). Both 2-PAMCl + atropine in combination with curcumin exhibited hepatoprotective and neuroprotective mechanisms

Thus the present study provides some interesting new observations for possible therapeutic combination upon exposure to DDVP. The study is one of the first few investigating the protective efficacy of curcumin with or without 2-PAMCl and atropine.
